# Identification of the *SAUR* Members in Woodland Strawberry (*Fragaria vesca*) and Detection of Their Expression Profiles in Response to Auxin Signals

**DOI:** 10.3390/ijms26083638

**Published:** 2025-04-11

**Authors:** Ruian Zhou, Jiahui Feng, Zhihong Zhang, Yuexue Liu

**Affiliations:** College of Horticulture, Shenyang Agricultural University, Shenyang 110866, China; zra_0222@163.com (R.Z.); feng@stu.syau.edu.cn (J.F.); zhang_sau@163.com (Z.Z.)

**Keywords:** strawberry, auxin signaling, *SAUR* gene family, fruit development

## Abstract

The *SAUR* (Small Auxin-Upregulated RNA) family members are important early auxin responsive genes in plants, playing a key regulatory role in the auxin metabolism, signal transduction, plant organ development, and abiotic stress response. Auxin signaling is also crucial for strawberry fruit development, but its specific regulatory mechanism remains unclear. In this study, bioinformatics methods were used to systematically identify and evaluate the *FvSAUR* gene family members associated with the auxin signaling in strawberry. The woodland strawberry Yellow Wonder line ‘YW5AF7’ was used as the material to further investigate the expressional characteristics of *FvSAUR* members in response to the auxin signals. A total of 64 members of the *SAUR* gene family were identified in the woodland strawberry genome, associated with *FvSAUR1-64*. Further bioinformatics analysis revealed that the *FvSAUR* members have undergone significant structural differentiation during evolution, and their encoded proteins exhibit diversity in folding stability, physicochemical properties, and other aspects. The prediction of the cis-elements in the promoter sequences of these genes suggests that the *FvSAUR* genes may mediate multiple hormonal and environmental signals, participating in a wide range of biological processes. RNA seq data analysis combined with RT-qPCR analysis revealed a dynamic spatiotemporal expression pattern of the *FvSAUR* genes in the vegetative and reproductive organs of strawberries, particularly the high expression levels of *FvSAUR11*, *17*, *19*, *21*, and other genes in flowers and young fruits, suggesting their potential regulatory roles in strawberry fruit development. Exogenous auxin treatment experiments further suggested that the expression of *FvSAUR11* and *FvSAUR19* is sensitive to the changes in auxin levels, indicating their potential involvement in auxin signal transduction during strawberry fruit development. Subcellular localization results showed that both proteins are located in the nucleus. The results of this study systematically analyzed the sequence structure characteristics, evolutionary history, expression patterns, and potential functions of the strawberry *FvSAUR* family members, providing important insights for further elucidating the roles of *FvSAUR* genes in strawberry fruit growth and development.

## 1. Introduction

As the auxin signal plays a significant role in the development of plants, auxin early-response genes also play a pivotal role in plant development. They are a unique set of genes that are rapidly modulated and activated when internal auxin concentrations change. Specifically, once the auxin signal is received in the cell nucleus, the auxin early-response genes immediately initiate their expression process. This mechanism ensures that plants can promptly respond to the fluctuations of the auxin signals and adjust their growth strategies accordingly [1]. These genes function as “information relay stations” throughout the auxin signal transduction process, situated downstream in the auxin signaling pathway, bridging the upstream signals with the downstream responses. Essentially, they serve as the switches, regulating various reactions in plant growth and developmental processes. Due to their pivotal role in connecting auxin signals with actual growth responses, they are also indispensable in the plant’s response to external environmental changes.

Aux/IAAs, GH3s, and SAURs are considered the three primary auxin early-response gene families [2,3]. Each family has its specific structural characteristics and functions. Notably, the SAUR family in plants contains more genes than the other families, reflecting its unique role in plant development. In fact, genes belonging to the SAUR family have been widely identified in various plants.

This began with the discovery of the *SAUR* genes in soybean hypocotyls by McClure and Guilfoyle [4]. As the research progressed, it was found that an increasing number of plant species contain gene members belonging to the SAUR family. This not only underscores the ubiquity of these genes but also broadens the scope for subsequent functional research and application. The *SAUR* family genes encode a class of plant-specific proteins, and these proteins perform vital functions in the process of plant growth, development, and response to environmental signal changes. Subsequent studies revealed that these gene family members exist in most plant species, such as *Arabidopsis*, corn, rice, sorghum, cotton, tomato, apple, lychee, potato, pepper, peach, citrus, and others [5,6,7,8,9,10].

The *SAUR* gene members play crucial roles in the various growth and developmental stages of different plants. For example, they are involved in the elongation of the hypocotyl, phototropic growth, the formation of apical hooks for adaptive growth, and leaf growth and senescence. More specifically, based on the existing research, the *SAUR* family genes can be classified into two main categories: those that promote growth and those that inhibit growth. For instance, genes like *AAM1*, *OsSAUR39*, and *OsSAUR45*, when overexpressed, can lead to the inhibition of plant growth, while others like *SAUR63* and *SAUR19-24* act as the growth activators, promoting plant growth [11]. Such a growth regulatory mechanism highlights the complexity and diversity of the *SAUR* gene family members in plant development.

Strawberry (*Fragaria* spp.), widely cultivated and consumed globally, is not only beloved for its enticing flavor and rich nutritional value but also has become a significant subject in scientific research. During the early stages of strawberry fruit development, the regulatory role of auxin signaling is particularly pronounced. Auxin plays a crucial role in cell division and elongation in fruit, directly influencing the morphological formation and maturation process of the fruit. In non-climacteric fruits like strawberry, variations in auxin concentration have a direct and profound impact on the fruit size, shape, and ultimate quality [12]. For instance, during the initial maturation stage of the strawberry fruit, auxin facilitates the increase in fruit volume by promoting cell expansion and division. As the fruit matures further, the concentration of auxin gradually decreases, which is closely associated with physiological changes as the fruit transitions from the growth phase to maturation.

Notably, the high-quality genome sequence of the wild-type woodland strawberry (*Fragaria vesca*) provides a solid foundation for the in-depth exploration of the gene family members and their functions in strawberry. As previously mentioned, the *SAUR* gene family plays a pivotal role in the auxin signal transduction. However, few reports have addressed the potential roles of SAUR genes in woodland strawberry fruit development, and there remains some ambiguity regarding the specific functions and mechanisms of action of these gene family members [13].

In light of this, our research has focused on the systematic identification and detection of the expression response of the *SAUR* gene family members in strawberry to the auxin signals. By integrating genomic structure, chromosomal location, and sequence analysis, we have successfully identified and characterized the members of the *SAUR* gene family in strawberry. The aim of this study is to deepen the understanding of the *SAUR* genes in strawberry and to provide a basic theoretical foundation for the future exploration of the specific roles of *FvSAUR* genes in the development of strawberry fruit, especially their roles in the auxin pathway.

## 2. Results

### 2.1. Sixty-Four SAUR Members Are Identified in Fragaria vesca

In the present study, we conducted a comprehensive bioinformatics analysis of the *Fragaria vesca* genome and identified 64 genes belonging to the *SAUR* family. These genes were consecutively designated as *FvSAUR1* to *FvSAUR64*, based on their chromosomal order. A detailed list of these genes, along with their relevant genomic information, can be found in Supplementary Table S1.

The amino acid lengths of the predicted *SAUR* proteins in *Fragaria vesca* displayed considerable variation, with their values ranging from 84 residues (in FvSAUR60) to 336 residues (in FvSAUR27). Correspondingly, the molecular weights of these proteins spanned between 9492.18 Da (FvSAUR60) and 40013.41 Da (FvSAUR27). From an isoelectric point (*pI*) perspective, the range extended from a low of 5.16 (FvSAUR46) to a high of 10.71 (FvSAUR29). These findings underscore the heterogeneity of SAUR proteins in strawberries, with some members exhibiting acidic properties while others lean toward basicity.

To delve deeper into the characteristics of the *FvSAUR* gene family members in strawberry, we analyzed several bioinformatics parameters, including their instability index, aliphatic index, and GRAVY. The instability index serves as a metric to predict the protein stability within a cellular context. Proteins with values above 40 are usually considered unstable. Notably, the majority of proteins encoded by the *FvSAUR* genes demonstrated that their indices are greater than 40, indicating their potential in vivo instability. FvSAUR1 and FvSAUR20 stood out with values over 60, suggesting their heightened instability. The aliphatic index, on the other hand, provides insights into the thermal stability of proteins. Proteins with elevated values are considered stable across an expansive temperature spectrum. Among the identified FvSAUR proteins, FvSAUR17 showed the highest aliphatic index, indicating its superb heat stability, while FvSAUR33 exhibited the lowest aliphatic index.

Lastly, the GRAVY index offers a glimpse into the hydrophilic or hydrophobic tendencies of a protein. Our observations revealed the varied GRAVY values across different FvSAUR proteins. Notably, FvSAUR9, FvSAUR12, FvSAUR17, FvSAUR45, FvSAUR46, FvSAUR47, and FvSAUR63 all possessed positive GRAVY values, suggesting that they may be hydrophobic.

### 2.2. Evolutionary and Structural Intricacies of the FvSAURs

**Figure 1:**
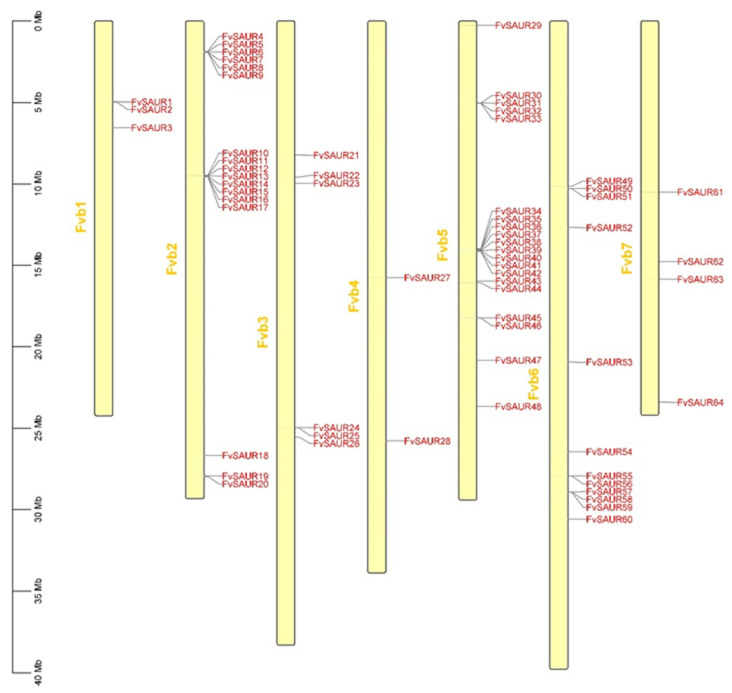
Chromosomal distribution map of the *SAUR* genes in *Fragaria vesca*. The vertical yellow bars with different lengths represent the chromosomes of *Fragaria vesca*, and the short black lines indicate the corresponding positions on the chromosomes of each gene.

The chromosomal distribution of these genes was initially ascertained. Our results (Figure 1) revealed that the *SAUR* members of *Fragaria vesca* were not evenly distributed across its chromosomes. Chromosome Fvb1 contains the genes *FvSAUR1* to *FvSAUR3*; chromosome Fvb2 harbors 17 genes ranging from *FvSAUR4* to *FvSAUR20*; chromosome Fvb3 consists of 6 genes from *FvSAUR21* to *FvSAUR26*; chromosome Fvb4 includes *FvSAUR27* and *FvSAUR28*; chromosome Fvb5 comprises 20 genes from *FvSAUR29* to *FvSAUR48*; chromosome Fvb6 contains 12 genes from *FvSAUR49* to *FvSAUR60*; chromosome Fvb7 encompasses 4 genes from *FvSAUR61* to *FvSAUR64*. The chromosome map illustrates the genomic distribution of these genes, which may provide clues to their evolutionary and functional relevance.

Furthermore, the inherent conserved motifs within the *FvSAUR* members play a pivotal role in the protein–protein interactions, as evidenced by their recurrent presence across various genes. All FvSAUR proteins contain the conserved structural domain of the Auxin Inducible Superfamily (cl23790), suggesting that the characteristic structural domains of the SAUR family of proteins are highly evolutionarily conserved.

**Figure 2:**
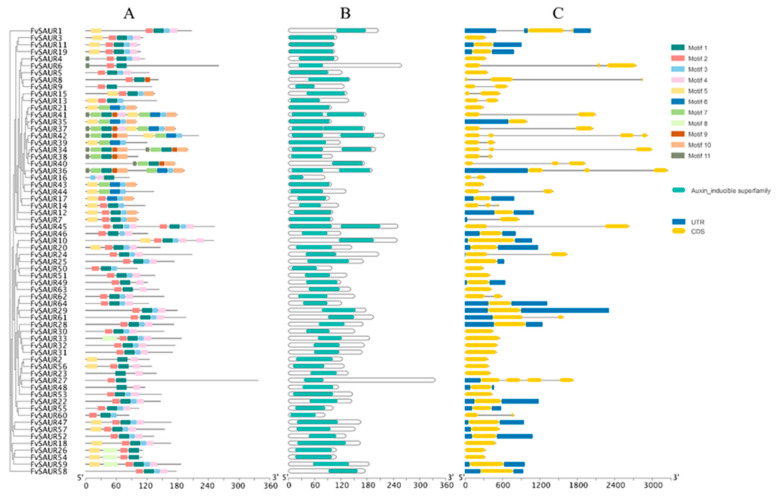
Comprehensive analysis of the *SAUR* gene family in *Fragaria vesca*: structural variability and conserved motifs. (**A**): Schematic representation of the conserved sequences of FvSAUR proteins. Blocks with different colors represent different conserved protein sequences; (**B**): locations of the conserved domains in each FvSAUR protein; (**C**): Exon/intron structure of *FvSAURs*. Yellow blocks represent exons, black lines represent introns, and blue blocks represent the untranslated regions (UTRs) at the 5′ or 3′ end.

The MEME [14] online tool was used to investigate the structural diversity of FvSAUR proteins in greater detail and to analyze their conserved motifs. A total of 11 conserved motifs were predicted (Figure 2), and all FvSAUR proteins were found to contain at least one or more of motif 1, motif 2, motif 3, and motif 4.

In addition, it was found that only a small subset of the *FvSAURs* contain introns, a structural characteristic that may be closely associated with gene expression regulation and functional diversity. Moreover, the length and number of introns vary among different genes, reflecting the structural and functional diversity of specific gene structures.

### 2.3. Phylogenetic Insights into the FvSAUR Gene Family: Unraveling Evolutionary Affiliations in Fragaria vesca

To elucidate the evolutionary lineage of the *FvSAUR* members in *Fragaria vesca*, we referenced the *AtSAUR* genes and constructed a phylogenetic tree based on the amino acid sequences encoded by these genes. The resulting unrooted phylogenetic tree demarcated distinct clusters, thereby highlighting the evolutionary relationships among these genes.

**Figure 3:**
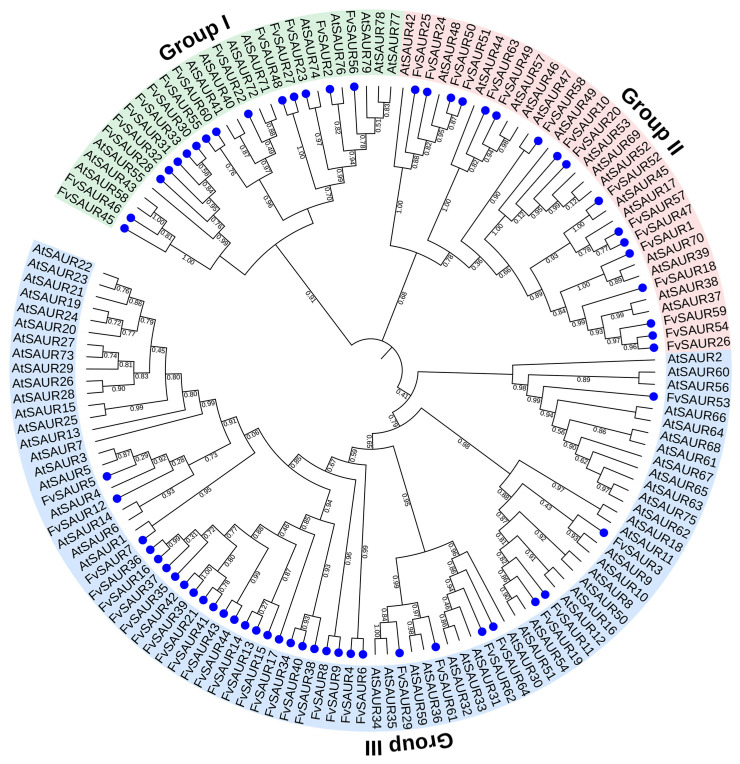
Phylogenetic analysis of the SAUR gene members in *Fragaria vesca*. AtSAURs of *Arabidopsis* serve as the references, and each FvSAUR is marked with a blue point.

Our phylogenetic tree analysis has revealed that these genes can be clustered into three main groups (Figure 3). Group 1 primarily includes the *Arabidopsis* SAUR genes *AtSAUR58*, *43*, *55*, *41*, *40*, *72*, *71*, *74*, *76*, *79*, *78*, *and 77* along with 15 FvSAUR genes: *FvSAUR45*, *46*, *28*, *32*, *31*, *33*, *30*, *55*, *60*, *22*, *48*, *27*, *23*, *2*, and *56*. Group 2 comprises *Arabidopsis* SAUR genes *AtSAUR42*, *48*, *44*, *57*, *46*, *47*, *49*, *53*, *69*, *52*, *45*, *17*, *70*, *39*, *38*, and *37*, in addition to 16 FvSAUR genes: *FvSAUR25*, *24*, *50*, *51*, *63*, *49*, *58*, *10*, *20*, *52*, *57*, *47*, *1*, *18*, *59*, *54*, and *26*. Group 3 is formed by the remaining genes in the phylogenetic tree, which include 33 *Fragaria vesca SAUR* genes.

High bootstrap values further substantiate the divisions within these groups. For example, the ensemble of *FvSAUR24*, *FvSAUR50*, and *AtSAUR48* indicates a pronounced evolutionary kinship among them, possibly suggesting a shared ancestral origin or similar gene functions. Analogously, additional clusters delineate relationships between the *FvSAUR* and *AtSAUR* genes, providing critical insights into the probable evolutionary dynamics of the *FvSAUR* gene family members when compared to the *AtSAUR* reference.

Within Group 2, the proximal clustering of genes like *FvSAUR25* and *FvSAUR54* suggests their shared evolutionary trajectory within the *SAUR* lineage. A notable observation in Group 3 is the distinct cluster formed by *AtSAUR60* and *AtSAUR2*, indicating a unique evolutionary lineage.

This classification not only reveals the potential evolutionary relationships among members of the *SAUR* gene family in woodland strawberry but also establishes the foundation for understanding their functional diversification. The genes within each group share a degree of sequence homology, which may suggest either functional similarities or evolutionary relatedness. In summary, this detailed phylogenetic analysis offers a comprehensive perspective of the evolutionary affiliations and tendencies inherent to the *FvSAUR* members, with *AtSAUR* genes serving as an insightful benchmark.

### 2.4. Analysis of the Cis-Acting Elements in FvSAURs Promoters

**Figure 4:**
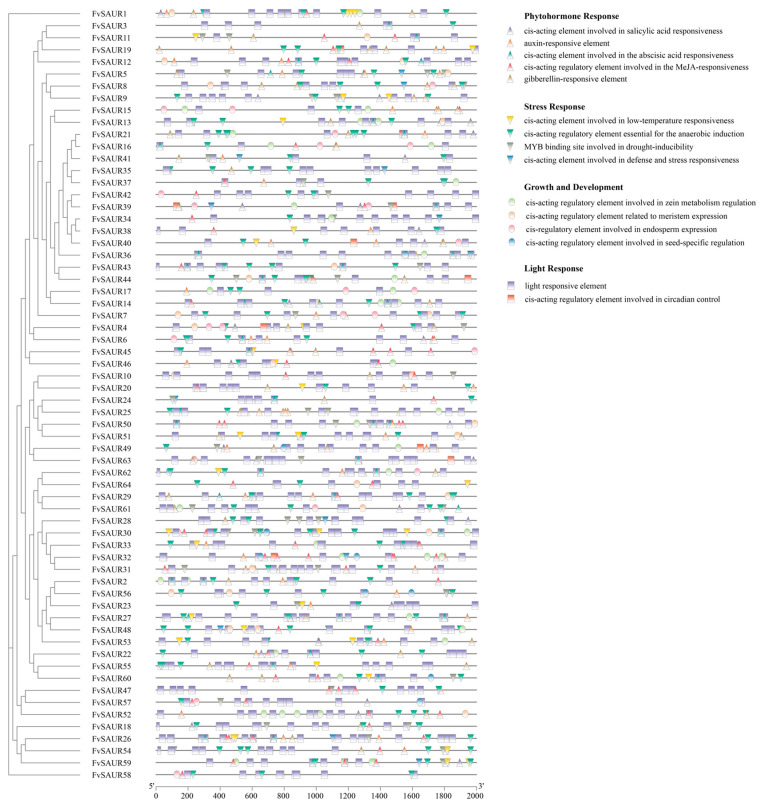
Distribution of cis-acting elements in promoters of the *FvSAURs*. Different symbols represent different cis-elements.

*Cis*-acting elements are critical modulators that dictate the magnitude and spatiotemporal framework of gene transcription. To characterize the cis-acting elements distributed in the *FvSAUR* promoter region, the 2000 bp sequences upstream of the initiation codon of each *FvSAUR* gene were extracted. Subsequent predictions of the cis-acting elements within the promoters were performed using the PlantCARE database. An abundance of light-responsive elements was identified, implicating their potential roles in the light-mediated signaling pathways. Intriguingly, a variety of hormone-responsive elements were also detected. For instance, *FvSAUR61* and 10 other *FvSAUR* members exhibited gibberellin-responsive elements (Figure 4). Similarly, *FvSAUR1* and *FvSAUR7* were found to harbor salicylic acid-responsive elements. Moreover, *FvSAUR30* and 11 additional members contain Jasmonic acid-responsive elements, while *FvSAUR25* and its corresponding genes possess abscisic acid-responsive elements. In addition, the promoter regions of *FvSAUR5*, *FvSAUR19*, and *FvSAUR54* were also found to contain growth hormone response elements. These data highlight the broad-spectrum involvement of the *SAUR* gene members in orchestrating hormone signaling cascades.

Additionally, the prediction pinpointed the cis-acting elements related to growth, development, and environmental stress adaptation. These encompass the endosperm-specific expression elements, meristematic tissue expression elements, elements conferring stress resistance, and elements responsive to cold temperatures.

In essence, the *SAUR* gene members in *Fragaria vesca* appear poised to modulate diverse growth, developmental processes, and stress responses, primarily through the intricate regulation of their associated cis-acting elements.

### 2.5. The Evolutionary Dynamics and Duplication Patterns of FvSAUR Genes

The duplication events within the *FvSAUR* gene members were discerned utilizing the MCSCAN analysis. Specifically, tandem duplication, segmental duplication, and whole-genome duplication events were all identified, which likely contributed to the expansion of the *SAUR* gene repertoire in *Fragaria vesca*.

To decipher the genetic interrelationships among *SAUR* members in *Arabidopsis thaliana*, tomato (*Solanum lycopersicum*), and *Fragaria vesca*, we performed a collinearity analysis across their respective genomes, focusing on the *SAUR* genes within *Fragaria vesca*.

**Figure 5:**
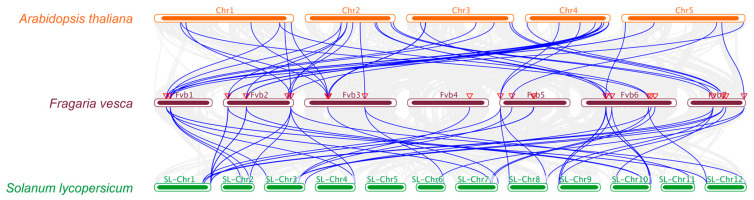
Collinearity and evolutionary insights into the *FvSAURs*. The collinearity of *SAUR* gene family members among woodland strawberry (*Fragaria vesca*), *Arabidopsis thaliana*, and tomato (*Solanum lycopersicum*) is shown. Genes connected by blue lines represent homologous genes.

The analysis revealed several notable gene pair associations. For instance, *FvSAUR1* was found to be collinear with *SlSAUR64* (Solyc07g042470), and *FvSAUR3* with *SlSAUR32* (Solyc02g062230) (Figure 5). Such pairings suggest that the selected *SAUR* genes might be evolutionarily conserved across disparate species.

The collinearity analysis further suggested the existence of 15 *FvSAUR* gene pairs that occupy collinear genomic blocks. Of particular interest, *FvSAUR3* displayed significant evolutionary dynamism, showing strong collinearity with two distinct tomato *SAUR* genes.

Intriguingly, our analysis revealed no evidence of singleton duplications or proximal duplications in the chromosomal regions containing *FvSAUR* genes. This absence implies that certain duplication mechanisms, such as whole-genome or segmental duplications, may have played a dominant role in the expansion of this gene family, while small-scale (singleton/proximal) duplications were either suppressed or selectively disadvantageous.

In summation, the *SAUR* gene lineage within *Fragaria vesca* expanded through distinct duplication events and retained critical functions throughout evolutionary time. This study provides insights into the complex evolutionary and functional dynamics of the *SAUR* gene members.

### 2.6. Different Expression Dynamics of FvSAUR Genes Across Developmental Stages and Tissues in Fragaria vesca

To elucidate the roles of the *FvSAUR* genes in *Fragaria vesca* development, we conducted a comprehensive investigation into their expression profiles, spanning the various developmental phases and the distinct tissue types of the strawberry, using the expression data, which were also obtained from the YW5AF7 line from the eFP database. Our meticulous analysis encompassed five distinct developmental stages of the cortex and pith of strawberry and delved into specialized tissues, including the microspore, perianth, receptacle, anther, whole flower, leaf, and seedling.

**Figure 6:**
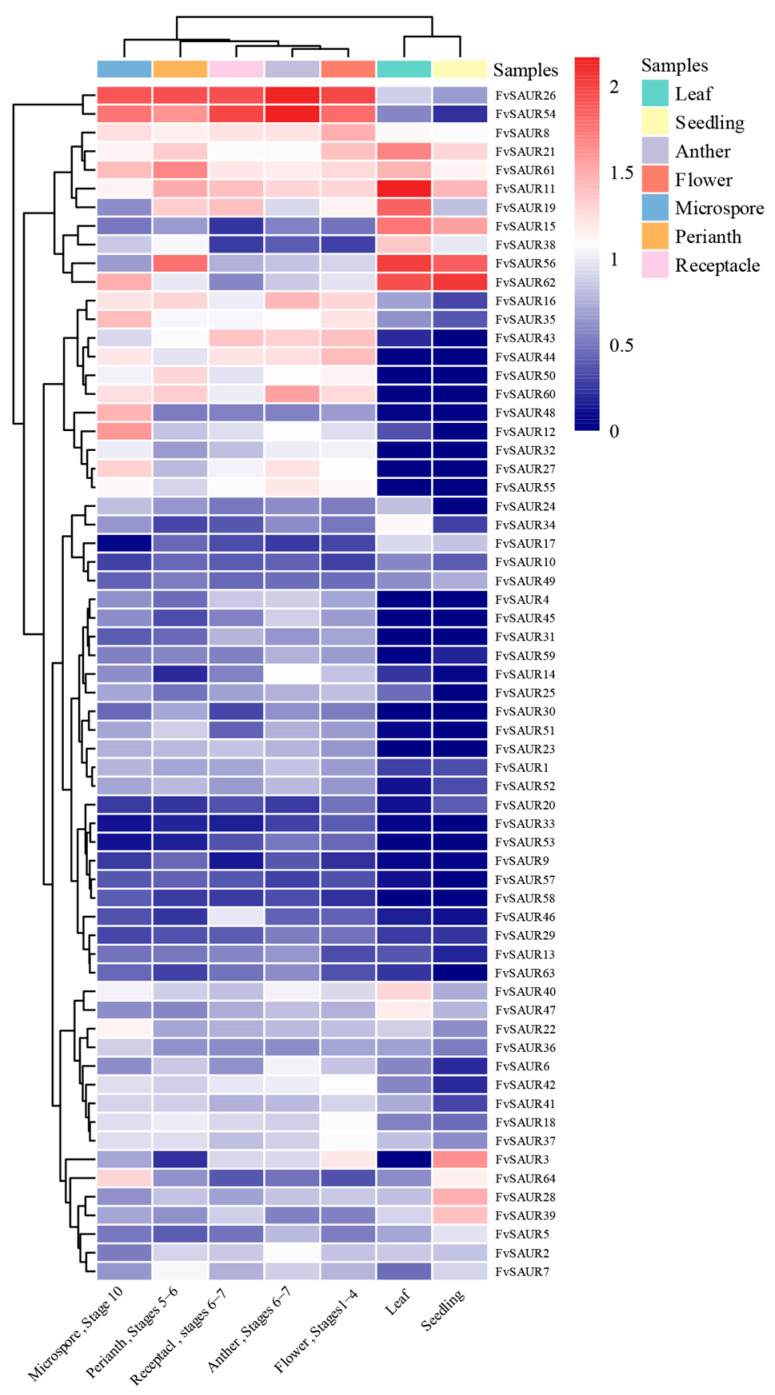
Expression patterns and developmental significance of the *FvSAUR* gene family in *Fragaria vesca*. Description of the stages for each sample could be found in Hawkins et al. (2017) [15].

Several prominent expression patterns were observed in their expression (Figure 6). Notably, *FvSAUR11*, *FvSAUR19*, *FvSAUR7*, *FvSAUR21*, *FvSAUR15*, and *FvSAUR38* exhibited strong expression levels in the pith and cortex of strawberry, consistently observed across all examined developmental stages. In contrast, genes such as *FvSAUR62*, *FvSAUR17*, *FvSAUR61*, *FvSAUR39*, and *FvSAUR56* showed higher expression during development stages 2 to 5 of the pith. It is noteworthy that *FvSAUR3*, *FvSAUR64*, *FvSAUR28*, and *FvSAUR39* were preferentially expressed during the seedling phase. Other members like *FvSAUR16*, *FvSAUR35*, *FvSAUR43*, and others were predominantly expressed in the microspore, perianth, receptacle, and anther but exhibited low expression levels in leaf and seedling tissues. Additionally, *FvSAUR15*, *FvSAUR38*, *FvSAUR56*, and *FvSAUR62* exhibited elevated expression in both the leaves and seedlings. Importantly, *FvSAUR8*, *FvSAUR21*, *FvSAUR61*, *FvSAUR11*, and *FvSAUR19* displayed pronounced expression levels across all tissue types investigated.

### 2.7. Differential Expression Patterns of FvSAUR Genes in Various Tissues: Insights from qRT-PCR Analysis

In order to further investigate whether *FvSAUR* gene members function in the early development of strawberry fruits, 10 gene members with significant expression in the medulla and cortex of strawberry fruits screened in the eFP database were further screened and analyzed by quantitative assays, including *FvSAUR2*, *FvSAUR7*, *FvSAUR11*, *FvSAUR15*, *FvSAUR17*, *FvSAUR19*, *FvSAUR21*, *FvSAUR54*, *FvSAUR61*, and *FvSAUR62*, based on the results of the heatmap analysis of pre-transcriptomic data. Notably, the expression trends revealed by quantitative PCR were consistent with the expression level changes shown in the RNA-seq heatmap.

**Figure 7:**
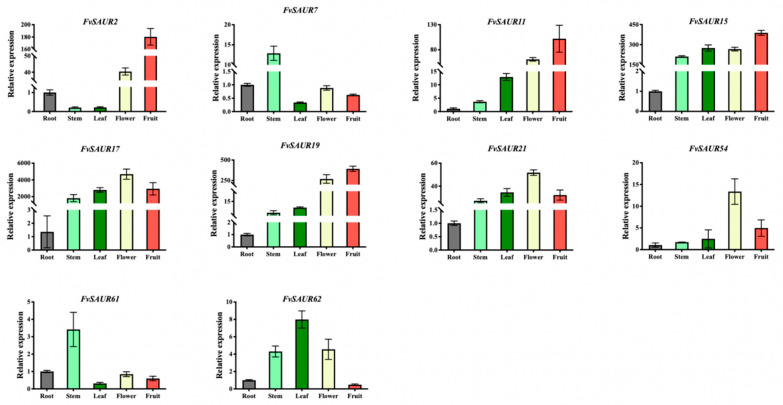
Differential expression patterns of *FvSAUR* gene members in various strawberry tissues revealed by RT-qPCR.

The expression patterns of these 10 selected genes in roots, stems, leaves, flowers, and fruits of the ‘YW5AF7’ strawberry cultivar at the same developmental period were first examined using real-time quantitative PCR (qRT-PCR). The quantitative PCR results (Figure 7) showed that *FvSAUR2* and *FvSAUR61* exhibited higher expression levels in leaves, and *FvSAUR62* also showed an elevated relative expression in leaves. In contrast, *FvSAUR17* and *FvSAUR54* displayed the highest relative expression in flowers. Meanwhile, *FvSAUR2*, *FvSAUR11*, *FvSAUR15*, and *FvSAUR19* all showed the most significant expression levels in fruits.

**Figure 8:**
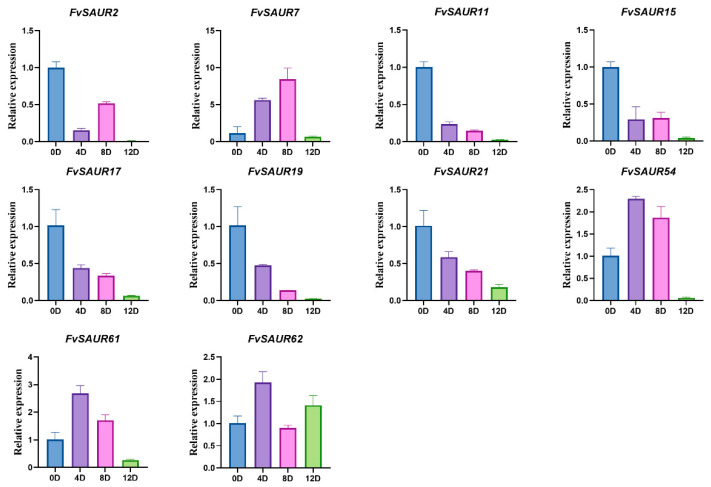
Expression of the *FvSAUR* gene in the early fruit of strawberry.

Considering the important roles of the auxin signal in the fruit development of strawberry, we further investigated the role of FvSAUR genes in early fruit development, and we also examined the expression changes in these genes at 0, 4, 8, and 12 days after pollination was detected. The results are shown in Figure 8; six genes (*FvSAUR2*, *FvSAUR11*, *FvSAUR15*, *FvSAUR17*, *FvSAUR19*, and *FvSAUR21*) exhibited their highest expression levels on the day of pollination (day 0). The expression levels of *FvSAUR11*, *FvSAUR17*, *FvSAUR19*, and *FvSAUR21* showed a decreasing trend as fruit development progressed. In contrast, the expression of the other nine genes, except *FvSAUR62*, was significantly higher on day 8 and before pollination compared to day 12.

### 2.8. Auxin Treatment Upregulates the Gene Expression of FvSAUR11 and FvSAUR19

Based on the expression patterns of *FvSAUR* genes in strawberry fruits at different developmental stages and the pooled expression data of these genes in the pith and cortex from the eFP database (Figure S1), we selected *FvSAUR11* and *FvSAUR19*, which showed a significant decreasing trend in early fruit development, for the exogenous auxin signal (NAA) and its transport inhibitor (NPA) treatment experiment to detect their expressional responses.

**Figure 9:**
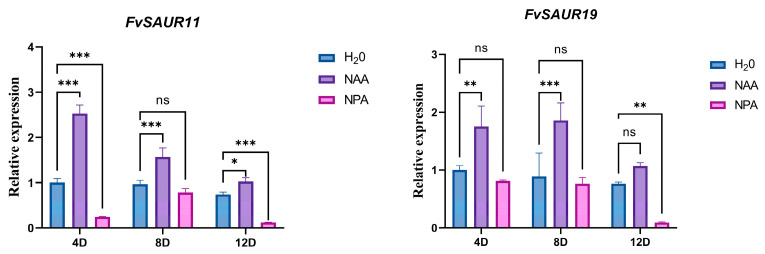
Effects of NAA treatment on the expression levels of *FvSAUR11* and *FvSAUR19*. Data were analyzed by two-way ANOVA; * *p* ≤ 0.033, ** *p* ≤ 0.002, *** *p* ≤ 0.001, and ns = 3.

As shown in Figure 9, the expression levels of both *FvSAUR11* and *FvSAUR19* were significantly increased at 4 and 8 days after NAA treatment, indicating that these two genes are clearly responsive to the auxin signaling. Conversely, the expression levels of these two genes were down-regulated after 12 days of NPA treatment.

### 2.9. FvSAUR11 and FvSAUR19 Are Subcellularly Located in the Nucleus

As noted, *FvSAUR11* and *FvSAUR19* show peak expression during the early strawberry fruit development stage and are both NAA-responsive, suggesting that they may function in the regulation of early fruit development. Most SAUR members function in plant development by regulating the transcription levels of target genes, indicating that SAURs should be transcriptional factors (TFs) localized in the nucleus. To further investigate this, we employed the GFP fusion protein technique to determine the precise cellular localization of the proteins encoded by these two genes.

**Figure 10:**
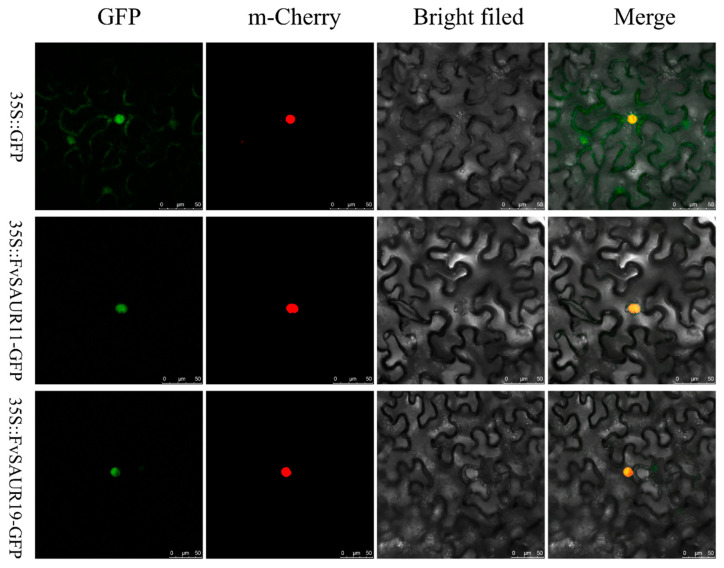
Subcellular localization of FvSAUR11 and FvSAUR19. m-Cherry serves as a reference marker, providing consistent red fluorescence in the nucleus.

As shown in the images (Figure 10), the first row serves as the control group, displaying widespread GFP expression with uniform green fluorescence throughout the entire cell, thereby establishing a baseline for a fluorescence background. Following this, the second and third rows illustrate the localization of the SAUR11 and SAUR19 GFP fusion proteins, respectively. The green fluorescence of these proteins reveals a localization pattern that is distinctly different from that of the control group, indicating a concentration of these SAUR proteins within the cell nucleus, which is consistent with their role as TFs.

## 3. Discussion

### 3.1. The SAUR Members of Woodland Strawberry

Woodland strawberry (*Fragaria vesca*) has become a model organism not only for the functional genomics research of strawberry but also for the studies on fruit development in horticultural crops. As SAUR members are the important factors that work in the process of auxin signal transduction, it is necessary and urgent to comprehensively analyze the structure, evolution and expression patterns of the SAUR members in *Fragaria vesca*. In this study, 64 members of the *SAUR* gene family were successfully identified in the woodland strawberry genome by bioinformatics analysis. The large number of SAUR members detected is consistent with previous reports in other plant species such as *Arabidopsis*, rice, tomato, etc., reaffirming the large size of the SAUR family in plant genomes [11,16].

Further biophysical characterization revealed that *FvSAUR*-encoded proteins exhibit significant differences in amino acid composition, the isoelectric point, and hydrophilicity. The diversity of physicochemical properties suggests that they may play varied roles in plant physiological and metabolic processes [17]. Notably, most FvSAUR proteins have high instability indices, indicating that they may function as short-lived regulators that can finely regulate the intensity and duration of growth hormone signaling through rapid degradation [11,18]. Such mechanisms enable plant cells to rapidly and dynamically sense changes in internal and external signals and make timely adaptive responses, which is particularly important under stress conditions.

In addition, phylogenetic analyses revealed that the *FvSAUR* genes can be divided into three major evolutionary branches, suggesting that they may have originated from a common ancestor but have undergone functional differentiation during long-term evolution. Interestingly, some *FvSAUR* genes are associated with repetitive sequences, such as retrotransposons, suggesting that transposon-mediated gene duplication may be one of the driving forces for the expansion of the SAUR gene family members [16]. Furthermore, the presence of multiple cis-acting elements in the promoter regions suggests that the *FvSAUR* genes may be extensively involved in plant growth and developmental regulatory networks by sensing and integrating multiple endogenous and exogenous signals. Gene covariance analyses further suggested that aneuploidy, as well as segmental duplications, are other important mechanisms for the evolutionary expansion of the FvSAUR gene family.

### 3.2. Spatiotemporal Expression Patterns of FvSAUR Genes Predict Their Diverse Physiological Functions

To further investigate the functions of the *FvSAUR* gene in the growth and development of strawberry, the present study systematically analyzed the dynamic expression patterns of *FvSAUR* genes in different tissues, organs, and fruits during the developmental period by combining RNA-seq data and real-time quantitative PCR. The results from both assays were highly consistent, which further supports the reliability of the data in this study. Notably, *FvSAUR12*, *19*, and *21* were dominantly expressed in flowers and fruits, and *FvSAUR17* transcripts were abundantly enriched in flowers, while *FvSAUR61* and *62* were mainly expressed in roots and stems. These findings suggested that different *FvSAUR* genes may exercise their respective tissue- and organ-specific functions through the fine spatiotemporal regulation of expression and collectively coordinating the processes of vegetative growth and reproductive development.

In fact, previous studies have shown that *AtSAUR19* plays a key role in hypocotyl elongation and growth in *Arabidopsis* [19]. *SlSAUR69* is involved in the expansion and growth of tomato fruits [20], and *OsSAUR39* plays an important role in another development in rice [21]. Therefore, the unique spatiotemporal expression pattern of *FvSAUR* genes revealed in this study likely predicts their extensive involvement in the regulation of multiple developmental stages of strawberry plants and fruits, which undoubtedly greatly expands the knowledge of the functional diversity of SAUR genes. Future studies should focus on specific *FvSAUR* genes to explore their molecular mechanisms and elucidate their key nodes in the complex regulatory network of strawberry growth and development in future work.

### 3.3. The Role of the Auxin Signal in Fruit Development

Previous studies have demonstrated that, in strawberry fruit, auxin primarily promotes transverse fruit expansion, whereas gibberellins (GAs) are more critical for longitudinal growth [22]. In this process, other phytohormones, such as cytokinin and ethylene, also play essential roles. During early fruit development, auxin, gibberellins, and cytokinins are present at high levels, stimulating cell division and expansion. Subsequently, ethylene biosynthesis is upregulated, facilitating fruit ripening. In auxin-mediated early fruit development, auxin-responsive genes play a pivotal role, with the *SAUR* gene family being a key representative. Studies have shown that the *SAUR* gene family modulates fruit shape and size by regulating both cell division and expansion [20]. Our results also demonstrated that the expression of both *FvSAUR11* and *FvSAUR19* decreased gradually with fruit development, and their expression was significantly induced by auxin treatment, while their transcription could be significantly inhibited by desexing and growth hormone signaling inhibitors, indicating that these two members belong to the typical auxin positively regulated genes, and their expression activities are closely related to endogenous growth hormone levels. In addition, promoter analysis revealed that pro*FvSAUR11* contains characteristic meristematic tissue *cis*-acting elements, while pro*FvSAUR19* is rich in growth hormone response motifs, providing possible molecular evidence for the hypothesis that they mediate the growth hormone signaling transduction involved in fruit development.

This study lays the groundwork for future research in which the biological functions of *FvSAUR11* and *FvSAUR19* will be further explored using genetic transformation and related experimental approaches. Moreover, our analysis revealed that their promoter sequences harbor stress-responsive cis-elements linked to low temperature and drought, implying potential involvement in abiotic stress responses. These putative functions will be further validated in future studies.

## 4. Materials and Methods

### 4.1. Identification and Validation of the FvSAUR Genes

In this study, genomic data of the woodland strawberry were derived from the latest woodland strawberry genome data v4.0.a2, which is available in the Genome Database for Rosaceae (GDR, https://www.rosaceae.org/, accessed on 10 October 2023). Given the model role of *Arabidopsis* in plant biology, its SAUR protein sequences served as an invaluable reference for the research. The initial step also involved the procurement of the *AtSAUR* gene sequences from the TAIR database (http://www.arabidopsis.org/, accessed on 10 October 2023). To ensure the authenticity and relevance of these sequences, we meticulously cross-referenced them with the records in the UniProt database (http://www.uniprot.org/, accessed on 15 October 2023).

The domain structures of these genes were further examined through the Pfam database (http://pfam.sanger.ac.uk, accessed on 10 December 2023). To reinforce the validation of the identified genes, we utilized the SAUR Hidden Markov Models (HMMs) from the Pfam database, analyzing them through the HMMER software v3.4, ensuring an e-value cutoff of 1 × 10^−10^ for the precise results.

### 4.2. Sequence and Structural Analysis

An in-depth exploration of the genetic structures of *FvSAURs* in strawberry was undertaken next. The exon/intron architectures and the specific chromosomal domiciles of *FvSAUR* genes were gleaned from the PLAZA (https://bioinformatics.psb.ugent.be/plaza/, accessed on 13 October 2023) and Phytozome v13 (https://phytozome-next.jgi.doe.gov/, accessed on 13 October 2023) databases. For further verification, we referred to the NCBI database (https://www.ncbi.nlm.nih.gov/). Employing the versatile TBtools software v2.019 [23], their intricate structures were visualized effectively.

On the protein front, the ExPASy (http://web.expasy.org/protparam/, accessed on 20 October 2023) was deployed to predict the crucial parameters, such as the molecular weight and the isoelectric point (pI) of the FvSAUR proteins. With an intent to uncover patterns, the MEME online tool (http://meme-suite.org/tools/meme, accessed on 20 October 2023) spotlighted the motifs within the protein sequences. The number of motifs was set to 11, and other parameters were set to default values. These motifs underwent a rigorous validation process through the Pfam (http://pfam.xfam.org/, accessed on 20 October 2023) database and the SMART server (http://smart.embl-heidelberg.de/, accessed on 8 April 2025). Additionally, with the aid of the CDD (Conserved Domain Database) platform of the NCBI (https://www.ncbi.nlm.nih.gov/, accessed on 20 October 2023), conserved domains within the proteins were predicted, offering insights into their potential functional areas.

### 4.3. Phylogenetic Analysis

To decipher the evolutionary relationships of the FvSAUR protein sequences, the AtSAUR proteins from *Arabidopsis* were included in the phylogenetic analysis. These gene sequences were meticulously aligned using the robust MAFFT v7.505 software [24]. Based on these alignments, phylogenetic trees were then constructed, employing the maximum likelihood method via the FastTree software v2.1.9 [25]. To facilitate an intuitive understanding of these findings, we visualized the tree using the user-friendly iTOL server (https://itol.embl.de/itol.cgi, accessed on 28 October 2023) [26].

### 4.4. Chromosomal Localization and Collinearity Analysis

Delineating the chromosomal roadmap of genes provides valuable evolutionary insights. Regarding this, the MapGene2 software v2.1 [27] was used to pinpoint the exact chromosomal abodes of *FvSAUR* genes. To widen our horizons, we procured genomic data from Phytozome (V13) spanning multiple species, including *F. vesca*, *A. thaliana*, and tomato. An in-depth collinearity analysis was then embarked upon using TBtools [23], the results of which were then presented in an accessible format via MCScanX’s Text Merge (http://chibba.pgml.uga.edu/mcscan2/, accessed on 15 December 2023) [28].

### 4.5. Promoter and Cis-Element Analysis

Promoter sequence analysis could provide valuable information about the regulation of gene expression. For this endeavor, promoter sequences (approximately 2000 bp upstream) specific to the strawberry *SAUR* genes were extracted from Phytozome (https://phytozome-next.jgi.doe.gov/, accessed on 23 October 2023). Using the PlantCARE database (https://bioinformatics.psb.ugent.be/webtools/plantcare/html/, accessed on 25 October 2023), the potential *cis*-elements of these genes, the crucial components in regulating gene expression, were identified and shown in Supplementary Table S2. These *cis*-elements were then subsequently visualized with clarity using TBtools [23], enabling a comprehensive analysis of the possible gene expression regulation.

### 4.6. Gene Expression Pattern Analysis

Expression patterns offer a direct window into the functional realm of genes. In this study, transcriptional abundance values of all *F. vesca SAUR* genes were sourced from the user-friendly strawberry electronic Fluorescent Pictograph (eFP) browser (https://bar.utoronto.ca/efp_strawberry/cgi-bin/efpWeb.cgi, accessed on 9 December 2023). This exhaustive transcriptional dataset, spanning various tissues and conditions, was finally elegantly visualized using TBtools, offering a holistic understanding of *FvSAUR* gene expression in different tissues and developmental stages.

### 4.7. Hormone Treatments

In this study, we focused on exploring the effects of the exogenous auxin signals on the *FvSAUR* gene expression changes and the early fruit development of woodland strawberry. As the strawberry fruit was developed from the receptacle following its expansion, the receptacle development changes and the *SAUR* gene expression changes under different auxin signal treatments were detected in this study.

The woodland strawberry Yellow Wonder line ‘YW5AF7’ grown in a greenhouse under natural conditions was selected, and flowers with petals just unfolded were set as 0 D for labeling and treatment. The hormone spraying dose was about 500 μM of each fruit each time. A small amount of skimmed cotton was wrapped around the receptacle and moistened with a syringe. The treatment was carried out once every 2 days, and the sampling time was 4 days, 8 days, and 12 days. The early receptacles of normal woodland strawberries were treated with NAA and NPA (N-1-naphthylphtha-lamicacid, inhibitor of the auxin signal transportation) after exogenously wrapping the cotton, and the control group was the early receptacles of normal forest strawberries sprayed with water.

### 4.8. RNA Isolation and Quantitative Real-Time PCR (qRT-PCR) Analysis

The samples used were frozen in liquid nitrogen immediately after collection and stored at −80 °C. The total RNA was extracted using the plant tissue RNA extraction kit (TIAN GEN, Beijing, China), and then the reverse transcription kit (TaKaRa, Dalian, China) was used to synthesize the cDNA for qRT-PCR experiments. qRT-PCR was performed using the SYBR Green PCR Master Mix Kit (TaKaRa, Dalian, China) on the CFX96 Real-Time PCR System (Applied Bio-systems, Foster City, CA, USA). Primer sequences are shown in Supplementary Table S3. Three individual samples were used for each treatment, and three biological replicate analyses were also performed.

### 4.9. Subcellular Localization

The subcellular localization assay was performed according to the method described in Yang et al. (2022) [29].

## 5. Conclusions

In this study, 64 *Fragaroa vesca SAUR* members were identified and characterized through systematic bioinformatics analysis. These *FvSAURs* showed a heterogeneous distribution on woodland strawberry staining, and bioinformatics analyses revealed their diverse molecular properties. *FvSAURs* showed dynamic spatiotemporal expression patterns in the vegetative and reproductive organs of woodland strawberry. Among these *FvSAUR* members, candidate genes with a high expression in early fruit development were identified, and further studies demonstrated that the expression levels of the *FvSAUR11* and *FvSAUR19* genes were sensitive to changes in auxin levels. Subcellular localization analysis suggested that FvSAUR11 and FvSAUR19 proteins are localized in the nucleus. This research provides the foundation for further studies of the strawberry *SAUR* family and establishes a framework for the identification and characterization of the *SAUR* genes in other species.

## Data Availability

The original contributions presented in this study are included in the article/Supplementary Materials. Further inquiries can be directed to the corresponding author.

## Supplementary Materials

The following supporting information can be downloaded at: https://www.mdpi.com/article/10.3390/ijms26083638/s1.
