# Supplementary material for: Identification of the SAUR Members in Woodland Strawberry (Fragaria vesca) and Detection of Their Expression Profiles in Response to Auxin Signals
Source: Int J Mol Sci. 2025 Apr 11;26(8):3638. doi: 10.3390/ijms26083638 (PMC12027354; doi:10.3390/ijms26083638)
Supplement: Supplementary file 1 [file ijms-26-03638-s001.zip › Supplementary Table S1.pdf]

Supplementary Table S1: Characterization of SAUR members in *Fragaria vesca*.

Name	Gene Locus ID	Number of amino acids	Molecular weight	Isoelectric Point(pI)	Instability index	Aliphatic index	GRAVY
FvSAUR1	FvH4_1g09280.t1	206	22661.37	6.31	67.47	68.16	-0.619
FvSAUR2	FvH4_1g09300.t1	124	13879.66	5.27	47.05	87.18	-0.303
FvSAUR3	FvH4_1g11980.t1	111	12646.56	7.72	46.59	90.45	-0.286
FvSAUR4	FvH4_2g02260.t1	114	12873.64	7.81	47.29	89.74	-0.237
FvSAUR5	FvH4_2g02280.t1	123	13947.13	6.58	38.63	95.12	-0.064
FvSAUR6	FvH4_2g02290.t1	259	30028.03	5.52	44.02	83.90	-0.497
FvSAUR7	FvH4_2g02300.t1	102	11202.99	8.76	17.50	98.53	-0.038
FvSAUR8	FvH4_2g02310.t1	141	16405.01	9.52	51.36	76.10	-0.349
FvSAUR9	FvH4_2g02320.t1	128	14773.47	10.12	56.09	97.42	0.075
FvSAUR10	FvH4_2g10760.t1	249	28790.57	9.95	57.80	99.40	-0.145
FvSAUR11	FvH4_2g10770.t1	105	12079.95	7.75	52.63	84.38	-0.358
FvSAUR12	FvH4_2g10800.t1	102	11234.00	9.25	55.77	95.49	0.084
FvSAUR13	FvH4_2g10810.t1	138	15544.91	9.52	52.90	74.93	-0.530
FvSAUR14	FvH4_2g10820.t1	115	13029.29	10.05	47.71	83.04	-0.289
FvSAUR15	FvH4_2g10850.t1	134	14946.11	6.06	43.79	80.00	-0.157
FvSAUR16	FvH4_2g10860.t1	84	9697.04	5.59	42.19	74.40	-0.520
FvSAUR17	FvH4_2g10870.t1	94	10368.09	6.55	33.19	103.83	0.146
FvSAUR18	FvH4_2g36430.t1	165	18874.63	8.8	46.67	72.06	-0.482
FvSAUR19	FvH4_2g38720.t1	106	12139.93	6.41	60.38	80.94	-0.397
FvSAUR20	FvH4_2g38740.t1	145	16536.01	9.78	68.08	78	-0.405
FvSAUR21	FvH4_3g13730.t1	99	11046.64	6.55	50.86	81.72	-0.229
FvSAUR22	FvH4_3g15390.t1	145	16165.32	9.34	68.56	92.07	-0.306
FvSAUR23	FvH4_3g15820.t1	137	15674.74	8.51	49.38	88.18	-0.524
FvSAUR24	FvH4_3g31140.t1	207	23512.11	7.68	39.19	88.5	-0.359
FvSAUR25	FvH4_3g31150.t1	172	19807.64	7.73	29.69	82.67	-0.571
FvSAUR26	FvH4_3g31390.t1	110	12397.55	10.57	36.08	87.82	-0.293
FvSAUR27	FvH4_4g12051.t1	336	40013.41	5.89	62.60	77.47	-0.806
FvSAUR28	FvH4_4g23230.t1	171	19065.96	9.32	47.89	71.17	-0.437
FvSAUR29	FvH4_5g00360.t1	178	20959.3	10.71	52.38	68.37	-0.681
FvSAUR30	FvH4_5g08770.t1	152	16933.64	8.51	38.11	76.91	-0.167
FvSAUR31	FvH4_5g08780.t1	169	18731.83	9.62	38.18	69.29	-0.392
FvSAUR32	FvH4_5g08790.t1	174	19468.51	9.3	47.57	75.11	-0.402
FvSAUR33	FvH4_5g08800.t1	186	20458.36	9.38	44.17	64.46	-0.411
FvSAUR34	FvH4_5g22620.t1	200	22653.91	5.39	40.79	82.8	-0.207
FvSAUR35	FvH4_5g22660.t1	99	10930.35	5.71	42.60	73.84	-0.297
FvSAUR36	FvH4_5g22690.t1	192	21715.76	5.85	36.03	81.72	-0.191
FvSAUR37	FvH4_5g22700.t1	175	19667.17	5.37	37.02	72.97	-0.357
FvSAUR38	FvH4_5g22780.t1	101	11185.66	5.27	43.99	76.34	-0.229
FvSAUR39	FvH4_5g22790.t1	119	13533.31	5.35	48.48	74.62	-0.473
FvSAUR40	FvH4_5g22810.t1	175	20074.09	9.56	53.19	75.26	-0.417
FvSAUR41	FvH4_5g22821.t1	178	19886.72	6.83	42.01	78.82	-0.230
FvSAUR42	FvH4_5g22822.t1	220	24933.33	5.86	35.45	82.00	-0.437
FvSAUR43	FvH4_5g24600.t1	99	10915.59	9.10	38.39	82.73	-0.083
FvSAUR44	FvH4_5g24720.t1	132	14879.16	9.38	40.33	76.14	-0.42
FvSAUR45	FvH4_5g26860.t1	251	28329.63	5.26	53.56	91.31	0.066
FvSAUR46	FvH4_5g26890.t1	120	13627.67	5.16	43.18	93.5	0.084
FvSAUR47	FvH4_5g29950.t1	166	18512.6	9.77	54.15	92.89	0.036
FvSAUR48	FvH4_5g32781.t1	115	13630.57	9.52	41.77	68.52	-0.918
FvSAUR49	FvH4_6g16110.t1	120	13750.03	9.03	33.15	82.83	-0.32
FvSAUR50	FvH4_6g16370.t1	100	11246.01	5.4	38.29	101.4	-0.21
FvSAUR51	FvH4_6g16380.t1	134	15275.53	6.22	33.85	84.4	-0.629
FvSAUR52	FvH4_6g19170.t1	132	14611.73	6.82	32.33	76.59	-0.146
FvSAUR53	FvH4_6g27350.t1	147	16707.5	9.86	63	97.48	-0.126

FvSAUR54	FvH4_6g33390.t1	110	12329.43	10.34	33.81	84.27	-0.325
FvSAUR55	FvH4_6g35400.t1	103	11919	9.51	27.38	79.32	-0.277
FvSAUR56	FvH4_6g35410.t1	128	14418.4	5.2	53.7	88.28	-0.23
FvSAUR57	FvH4_6g36840.t1	153	17351.15	8.73	28.04	85.95	-0.19
FvSAUR58	FvH4_6g36850.t1	176	20307.28	8.83	57.32	88.07	-0.507
FvSAUR59	FvH4_6g36860.t1	185	20800.98	9.11	44.11	77.46	-0.338
FvSAUR60	FvH4_6g38650.t1	84	9492.18	9.06	36.51	91.55	-0.292
FvSAUR61	FvH4_7g11280.t1	195	21742	9.85	33.64	64.97	-0.586
FvSAUR62	FvH4_7g17340.t1	152	17757.13	6.23	30.76	81.91	-0.612
FvSAUR63	FvH4_7g19120.t1	143	16767.86	9.16	39.53	91.26	0.058
FvSAUR64	FvH4_7g32600.t1	122	13979.89	6.19	36.36	71.89	-0.493
