# Supplementary material for: Identification of the SAUR Members in Woodland Strawberry (Fragaria vesca) and Detection of Their Expression Profiles in Response to Auxin Signals
Source: Int J Mol Sci. 2025 Apr 11;26(8):3638. doi: 10.3390/ijms26083638 (PMC12027354; doi:10.3390/ijms26083638)
Supplement: Supplementary file 1 [file ijms-26-03638-s001.zip › Supplementary Table S3.pdf]

Supplementary Table S3: Primer sequences used in qRT-PCR.

Gene ID	primer sequence/F	primer sequence/R
FvSAUR2	TTTCAAGGAGCTGGCAGAGA	GAGCTCGTTCATCGATTCCG
FvSAUR7	AGCTCATGCCAAGCAGAAAC	ATGGCCTTTCGGAACATCAA
FvSAUR11	GCTTCGATCACGACATGGG	AGCGTGGAAGTTAGAGAACGA
FvSAUR15	ACATGGGTTTTCGGTTACCA	TCTTCTGCTTGCCTCAACAA
FvSAUR17	TGTTGGGAAGAGCCAGAAAA	GAAGGCGTCTTCACTACATGG
FvSAUR19	GGGAAGAAGCAGCAACACTA	TCGGGTCAGGAAAGAGATGG
FvSAUR21	ATGGAAACCAGACAGCTCCA	GGGATTGTGATACCGCCCAT
FvSAUR54	GCAAATGCCCCAAAGAATAA	AAACTGCTTGAGCTCCTTGC
FvSAUR61	TCTGGTGCCCGTGATTTACT	CCGTATTCTTGCTCGGCTTC
FvSAUR62	TACGGGTTCGATCAGAAGGG	CTGAACGTACCGGAACTCCT
