# Supplementary figures and images for: Identification of the SAUR Members in Woodland Strawberry (Fragaria vesca) and Detection of Their Expression Profiles in Response to Auxin Signals
Source: Int J Mol Sci. 2025 Apr 11;26(8):3638. doi: 10.3390/ijms26083638 (PMC12027354; doi:10.3390/ijms26083638)

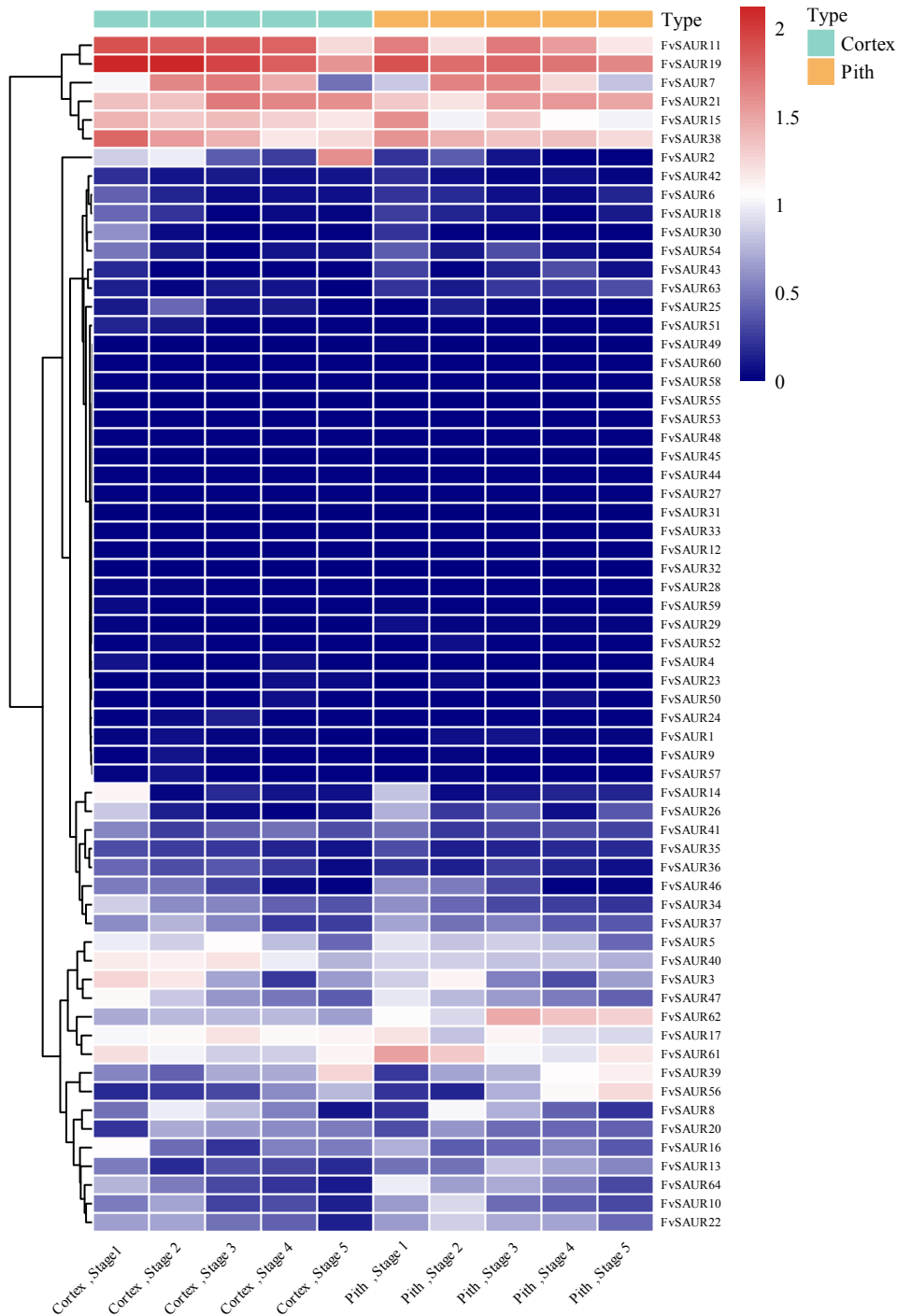

Supplement: Supplementary file 1 [file ijms-26-03638-s001.zip › Supplementary Figure S1.pdf]
